# Short-duration migraine without aura in children: a retrospective cohort study of attack duration and prognosis

**DOI:** 10.3389/fneur.2026.1782182

**Published:** 2026-03-10

**Authors:** Jiajun Ma, Zhiwei Yu, Xin Li, Tianyi Li, Li Jiang

**Affiliations:** Department of Neurology, Children’s Hospital of Chongqing Medical University, National Clinical Research Center for Children and Adolescents’ Health and Diseases, Ministry of Education Key Laboratory of Child Development and Disorders, International Science and Technology Cooperation Base of Child Development and Critical Disorders, Chongqing Key Laboratory of Child Neurodevelopment and Cognitive Disorders, Chongqing, China

**Keywords:** attack duration, children, follow-up, headache prognosis, migraine without aura, retrospective cohort, short-duration migraine

## Abstract

**Background:**

Migraine without aura (MwoA) is the most common migraine subtype in children and can cause persistent impairment of quality of life. The International Classification of Headache Disorders, 3rd edition (ICHD-3), requires an attack duration of ≥2 h to diagnose pediatric MwoA; however, this threshold is debated, and several studies suggest that a lower limit may be appropriate in children.

**Methods:**

In this single-center retrospective study, children with primary headache seen in our neurology clinic from 2018 to 2023 were identified. Baseline demographic and headache characteristics were extracted from records. Patients were grouped by typical attack duration into a short-duration MwoA group (SdMwoA, ≥1 min to <2 h) and an MwoA group. Headache outcomes at 6 months, 12 months and at the last follow-up were collected via clinic visits or structured telephone interviews. Multivariable logistic regression was used to identify factors associated with headache-free status and any improvement at 6 and 12 months.

**Results:**

Of the 293 screened children, 192 (97 SdMwoA patients and 95 MwoA patients) met the inclusion criteria; follow-up data were available for 67 (69.1%) and 66 (69.5%) patients, respectively. Compared with the SdMwoA group, the MwoA group more often had a headache history ≥1 month before presentation (61.1% vs. 41.2%, *p* = 0.006) and prior preventive treatment (29.5% vs. 12.4%, *p* = 0.004), while the other baseline features were similar. At the last follow-up, headaches had remitted or improved in 57/67 (85.1%) SdMwoA patients and 54/66 (81.8%) MwoA patients (*p* = 0.613). Typical attack duration was not associated with outcomes according to multivariate regression modeling.

**Conclusion:**

SdMwoA and MwoA have comparable short- to mid-term outcomes. These findings support a more flexible, child-centered interpretation of the ICHD-3 duration criterion in pediatric migraine, indicating that typical migraine phenotypes with attacks less than 2 h warrant consideration of MwoA when alternative diagnoses have been excluded.

## 1 Introduction

Migraine is among the most common primary headache disorders in children and adolescents and is characterized by recurrent headache attacks, school absenteeism, and frequent medical visits, imposing a sustained burden on patient quality of life and family caregiving (1). Among its clinical subtypes, migraine without aura (MwoA) is the most prevalent, accounting for approximately 70–80% of all migraine cases (2). According to the International Classification of Headache Disorders, 3rd edition (ICHD-3), migraine without aura (MwoA) is defined by (A) at least five attacks; (B) attacks lasting 4–72 h (2–72 h in children and adolescents); (C) headache with at least two of the following characteristics: unilateral location (often bilateral in children and adolescents), pulsating quality, moderate to severe pain intensity, and aggravation by routine physical activity; (D) during headache, at least one of the following: nausea and/or vomiting, photophobia, or phonophobia; and (E) the attacks are not better accounted for by another diagnosis (3). Although the ICHD-3 has introduced pediatric-specific adjustments, its applicability to pediatric populations remains a matter of debate, particularly regarding the appropriateness of the “2–72 h” duration criterion (4).

In pediatric headache clinics, clinicians frequently encounter a subgroup of children whose headaches are characterized by recurrent, moderate-to-severe, pulsating pain accompanied by typical migraine-associated symptoms such as nausea, vomiting, photophobia, and phonophobia. Their clinical phenotype is highly consistent with that of MwoA; however, the duration of individual attacks is markedly shorter than the lower limit of 2 h specified by the ICHD-3 (5), and in some patients, the headache lasts only tens of minutes (6, 7). Under the current diagnostic framework, these patients are typically classified as having “probable migraine without aura (probable MwoA)” and are therefore excluded from a definite diagnosis of MwoA (3). Francis et al. proposed the concept of “brief migraine episodes” to describe children with a typical migraine phenotype but an attack duration clearly shorter than the ICHD-defined minimum. However, that study focused primarily on baseline clinical characteristics and diagnostic sensitivity and did not compare follow-up prognosis between brief migraine episodes and MwoA (7). Overall, both that work and most previous prospective studies of pediatric MwoA have concentrated on baseline clinical phenotypes and the sensitivity and specificity of diagnostic criteria. Children with a typical migraine phenotype but short attack duration have often been excluded, and there is a lack of outcome-oriented, systematic analyses based on longitudinal follow-up data (8).

To address this evidence gap, the present study adopts follow-up outcomes as the primary evaluative framework. We hypothesized that if children with a typical migraine phenotype but attack duration <2 h show broadly comparable remission rates, cumulative proportions achieving a headache-free state, and associated prognostic factors compared with children whose MwoA attacks last ≥2 h, then excluding the former solely on the basis of attack duration may not be justified. Conversely, if the two groups exhibit clearly different prognostic patterns, short-duration cases may warrant distinct clinical consideration within the current diagnostic framework.

Accordingly, in this study, we enrolled consecutive pediatric patients who were admitted to the headache specialty clinic of a tertiary children’s hospital whose clinical phenotype was consistent with that of patients with migraine without aura. Patients were stratified according to the typical duration of individual attacks into two groups: those with attack durations ≥1 min and <2 h and those with durations ≥2 h and <72 h. Baseline clinical characteristics were retrospectively extracted, and headache outcomes were recorded at 6 months, 12 months, and the last follow-up. We compared remission rates and the cumulative proportion of children who achieved a headache-free state across different attack duration groups and further explored the factors associated with follow-up outcomes. The overarching aim of this study was to evaluate, from a prognosis-oriented perspective, the real-world clinical significance of single-attack duration in pediatric migraine without aura and to provide pediatric evidence on whether the “duration criterion” for MwoA in the ICHD-3 warrants conditional review.

## 2 Methods

### 2.1 Study design and ethics approval

This was a single-center retrospective cohort study conducted at the Children’s Hospital of Chongqing Medical University (Chongqing, China) between 1 January 2018 and 31 December 2023. Eligible participants were children who presented to the Department of Neurology with headache and met the predefined inclusion criteria.

The study is reported in accordance with the STROBE guidelines for observational research, and the protocol was approved by the Ethics Committee of the Children’s Hospital of Chongqing Medical University (Approval No. 2025-453). Because of the retrospective design and use of deidentified data, the requirement for written informed consent was waived. For children who required telephone follow-up, the research staff explained the study objectives to the guardians and obtained verbal consent before the interview. All the data were fully deidentified prior to analysis to ensure participant privacy.

### 2.2 Participants

To ensure consistency in case ascertainment, two pediatric neurologists with experience in pediatric headache independently and blindly reviewed the deidentified medical records. Any discrepancies were resolved through discussion until a consensus was reached.

The inclusion criteria were as follows: (1) age 4–18 years; (2) presentation to the neurology outpatient clinic for primary headache, with a standardized neurological examination and, when clinically indicated, ancillary investigations (e.g., neuroimaging) to exclude secondary headache; and (3) a diagnosis of migraine without aura (MwoA) or short-duration MwoA (SdMwoA) according to ICHD-3 as defined below (3). Photophobia/phonophobia were abstracted from the index-visit documentation based on patient/caregiver self-report and/or clinician-recorded behavioral indicators (e.g., light/sound avoidance).

The exclusion criteria were as follows: (1) evidence or strong suspicion of secondary headache, including but not limited to intracranial structural lesions, central nervous system infections, cerebrovascular disease, or metabolic/toxic disorders; (2) medication overuse headache (MOH) as defined by the ICHD-3 or medication use in the 3 months prior to presentation that could not be ruled out as having a substantial effect on attack duration; (3) short-lasting entities (e.g., seconds-long stabbing pains without migraine-associated symptoms, attacks predominantly triggered by cough or exertion), which were excluded to avoid misclassifying very brief attacks; (4) the absence of any migraine-associated symptom (nausea/vomiting and/or photophobia/phonophobia) documented at the index visit and/or headache features more consistent with tension-type headache (TTH); and (5) the absence of a determinable typical duration of individual attacks in the baseline record or clearly conflicting duration records that precluded reliable group allocation.

All eligible children were stratified by the typical duration of individual attacks into two groups: (1) SdMwoA: patients who met all MwoA criteria except attack duration (criterion B), with a typical attack duration ≥1 min and <2 h, no other pediatric adaptations were applied. A pragmatic lower boundary of ≥1 min was used to support reliable ascertainment in retrospective documentation and to reduce inclusion of extremely fleeting pains more typical of other short-lasting headache disorders. (2) MwoA: patients who fully met the ICHD-3 criteria A–E for MwoA (3). For all group assignments, the baseline record had to contain a clear description of the duration of individual attacks. If duration was documented as a range, grouping was based on the most reported typical attack duration.

### 2.3 Data collection

The following baseline data were extracted from the electronic medical records at the index visit: (1) demographic characteristics: age and sex; (2) headache clinical features: duration of individual attacks, headache history prior to presentation, headache location, laterality, headache quality, attack frequency and pain intensity assessed using a 0–10 numeric rating scale and classified as mild, moderate, or severe; (3) past history and comorbidities: emotional or psychological disorders and other chronic pain conditions; (4) family history of headache: at least one first-degree relative diagnosed with migraine; (5) pre-visit preventive treatment: any migraine preventive therapy initiated before the index visit and documented in the baseline record; and (6) functional disability: headache-related disability quantified using the Pediatric Migraine Disability Assessment Scale (PedMIDAS) and graded as 0–10 (little to none), 11–30 (mild), 31–50 (moderate), and >50 (severe) (9).

Follow-up data were obtained from outpatient revisit notes or from structured telephone interviews conducted by trained research personnel using a standardized template. The prespecified follow-up windows corresponded to headache durations of 6 months (±4 weeks) and 12 months (±4 weeks). The last follow-up was defined as the last available effective follow-up record. When both outpatient and telephone data were available for the same time window, the outpatient records were used. If the first follow-up attempt failed, at least three additional contact attempts were made on different days or at different times of day.

Headache outcomes at each follow-up were categorized according to the following predefined criteria: remission, defined as the complete absence of any headache attacks for ≥4 consecutive weeks with no emergency visits or records of rescue medication use; improved, defined as a ≥50% reduction in headache frequency or intensity compared with baseline, sustained for ≥4 weeks; stable, defined as no obvious change compared with baseline or some improvement that did not reach the threshold for improved; and worsened, defined as an increase in headache frequency, intensity, or functional impairment compared with baseline. On the basis of these categories, outcomes were further dichotomized as headache-free (remission) and any improvement (remission or improved).

### 2.4 Statistics

An *a priori* sample size calculation was not performed owing to the retrospective design based on existing medical records. The two primary endpoints were headache-free status and any improvement at 6 and 12 months. Categorical variables are summarized as counts and percentages. Continuous variables with an approximately normal distribution are presented as the mean ± standard deviation (SD), whereas skewed variables are summarized as the median and interquartile range (IQR). Depending on the data type and distribution, between-group comparisons were performed using the chi-square test or Fisher’s exact test for categorical variables and two-sided unpaired *t*-tests or Mann–Whitney *U* tests for continuous variables.

Children with incomplete baseline information that precluded reliable eligibility assessment or group allocation were excluded from the analytic cohort. For outcome analyses at each time point, analyses were conducted using complete-case data at each time point/model; no imputation was performed. Missing follow-up observations were treated as loss to follow-up. To evaluate the associations between attack duration and headache outcomes at different time points, we first performed univariable logistic regression analyses. Candidate predictors included short-duration attacks, sex, age, headache history ≥1 month prior to presentation, pre-visit preventive treatment, family history of headache, dizziness, comorbid chronic pain, self-rated anxiety and/or depression, unilateral headache, pain location, pain quality, attack frequency, moderate or severe pain, and PedMIDAS disability grade.

Focusing on the two predefined primary time points (6 and 12 months), we then constructed four multivariable logistic regression models, using headache-free status and any improvement at each time point as dependent variables. The attack-duration group was forced into all multivariable models. Variables with *p* < 0.10 in univariable analyses, together with baseline variables that differed meaningfully between groups or were considered potential confounders on the basis of clinical judgment, were entered into the multivariable models. Backward stepwise selection based on likelihood ratio tests was applied, and adjusted odds ratios (ORs) with 95% confidence intervals (95% CIs) were reported. To reduce time-related bias, children whose headache history at baseline already exceeded 6 months were excluded from analyses of 6-month outcomes, and those whose headache history exceeded 12 months were excluded from analyses of 12-month outcomes. The results of the multivariable logistic regression models are displayed as forest plots to visually compare the relative impact of different predictors on prognosis.

To test the robustness of our primary conclusions, we prespecified and conducted the following sensitivity analyses: (1) we repeated the multivariable logistic regression analyses under two extreme assumptions, in which all patients lost to follow-up were assumed either to have achieved the outcome or not to have achieved the outcome; and (2) if the SdMwoA and MwoA groups differed significantly in baseline characteristics other than attack duration, we further subdivided the short-duration group into 1–30 min and 30 min–2 h and reran the models to evaluate the impact of different short-duration strata on outcomes.

In addition, using the time from onset of index migraine attack as time zero, we applied the Kaplan–Meier method to estimate the cumulative probability of achieving a headache-free state during follow-up and compared survival curves between the SdMwoA and MwoA groups using the log-rank test. For children who did not become headache free during the study period but whose follow-up data were available, survival time was censored at the date of the last follow-up. For individuals with exceptionally long follow-up durations, survival time was censored at 2,000 days to avoid the undue influence of extreme values on the model. All the statistical analyses were performed using R version 4.3.1 (R Foundation for Statistical Computing, Vienna, Austria). A two-sided *p*-value <0.05 was considered to indicate statistical significance.

## 3 Results

### 3.1 Enrollment and grouping of participants

**Figure 1:**
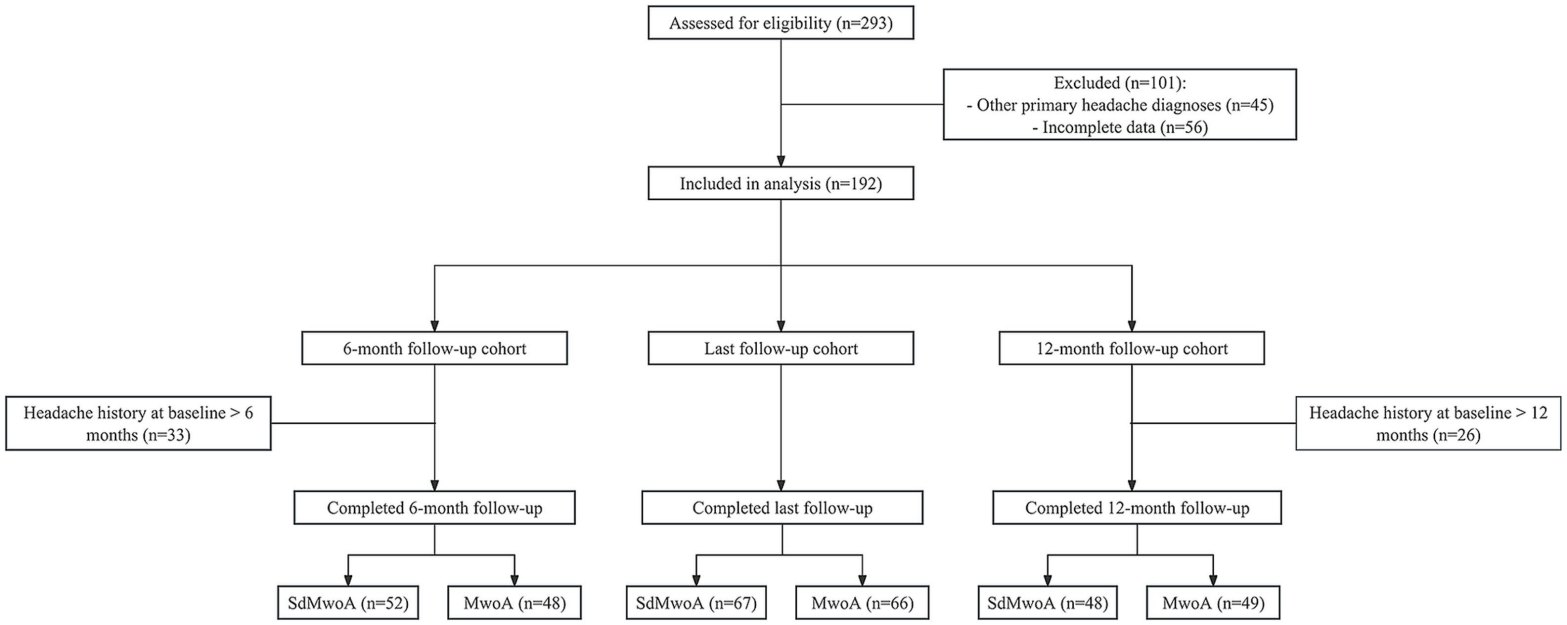
Flow chart of patient selection, grouping, and follow-up availability.

During the study period, 293 children presenting for an initial evaluation of primary headache were screened. Of these, 101 were excluded because of a diagnosis of other primary headache disorders (*n* = 45) or incomplete baseline (*n* = 56), leaving 192 children who were included in the analyses. On the basis of the typical duration of individual attacks, 97 patients were classified into the SdMwoA group, and 95 were classified into the MwoA group. Follow-up data at 6 months were available for 52/82 (63.4%) children in the SdMwoA group and 48/77 (62.3%) in the MwoA group; at 12 months, follow-up data were available for 48/86 (55.8%) and 49/80 (61.3%), respectively. For the final outcome analysis, the last follow-up data were available for 67 children in the SdMwoA group and 66 in the MwoA group (Figure 1).

### 3.2 Baseline clinical characteristics

The baseline clinical characteristics of the two groups are summarized in Table 1. Overall, the SdMwoA and MwoA groups had comparable distributions of sex, age, headache location, laterality, pain quality, attack frequency, and pain intensity, with none of these core headache-phenotype variables differing significantly between groups. There were also no significant between-group differences in family history of headache, coexisting chronic pain conditions, self-reported anxiety or depression, or PedMIDAS disability grade.

**Table 1:** Baseline characteristics of participants.

Variables	SdMwoA (*n* = 97)	MwoA (*n* = 95)	*p*-value
Sex, *n* (%)			0.677
Female	40 (41.2%)	42 (44.2%)	
Male	57 (58.8%)	53 (55.8%)	
Age, years, median (IQR)	10.0 (8.0, 11.0)	10.0 (8.0, 12.0)	0.079
Headache history ≥1 month, *n* (%)	40 (41.2%)	58 (61.1%)	**0.006**
Pre-visit preventive treatment, *n* (%)	12 (12.4%)	28 (29.5%)	**0.004**
Family history of headache, *n* (%)	20 (20.6%)	22 (23.2%)	0.670
Dizziness, *n* (%)	26 (26.8%)	36 (37.9%)	0.100
Other chronic pain, *n* (%)	32 (33.0%)	29 (30.5%)	0.714
Self-rated anxiety and/or depression, *n* (%)	10 (10.3%)	7 (7.4%)	0.473
Pain laterality, *n* (%)			0.673
Bilateral	43 (44.3%)	45 (47.4%)	
Unilateral	54 (55.7%)	50 (52.6%)	
Pain location, *n* (%)			0.147
Frontal	30 (30.9%)	16 (16.8%)	
Mixed	37 (38.1%)	45 (47.4%)	
Occipital	3 (3.1%)	5 (5.3%)	
Temporal	18 (18.6%)	23 (24.2%)	
Vertex	9 (9.3%)	6 (6.3%)	
Pain quality, *n* (%)			0.943
Other	78 (80.4%)	76 (80.0%)	
Throbbing	19 (19.6%)	19 (20.0%)	
Pain frequency, *n* (%)			0.446
<1/month	5 (5.2%)	4 (4.2%)	
1–3/month	5 (5.2%)	6 (6.3%)	
1/week	6 (6.2%)	8 (8.4%)	
2–6/week	16 (16.5%)	19 (20.0%)	
Daily	65 (67.0%)	58 (61.1%)	
Pain intensity, *n* (%)			0.191
Mild	14 (14.4%)	8 (8.4%)	
Moderate or severe	83 (85.6%)	87 (91.6%)	
PedMIDAS^a^, *n* (%)			0.236
Little to none	16 (16.5%)	7 (7.4%)	
Mild	36 (37.1%)	40 (42.1%)	
Moderate	18 (18.6%)	17 (17.9%)	
Severe	27 (27.8%)	31 (32.6%)	

a PedMIDAS: Pediatric Migraine Disability Assessment Scale graded as 0–10 (little to none), 11–30 (mild), 31–50 (moderate), and >50 (severe).

Bold values indicate statistically significant between-group differences (two-sided *p* < 0.05).

In contrast, a greater proportion of children in the MwoA group than in the SdMwoA group had a headache history of at least 1 month before presentation (61.1% vs. 41.2%, *p* = 0.006), and children in the MwoA group were also more likely to have received pre-visit preventive treatment before the index visit (29.5% vs. 12.4%, *p* = 0.004).

### 3.3 Headache outcomes at 6 months, 12 months, and last follow-up

**Figure 2:**
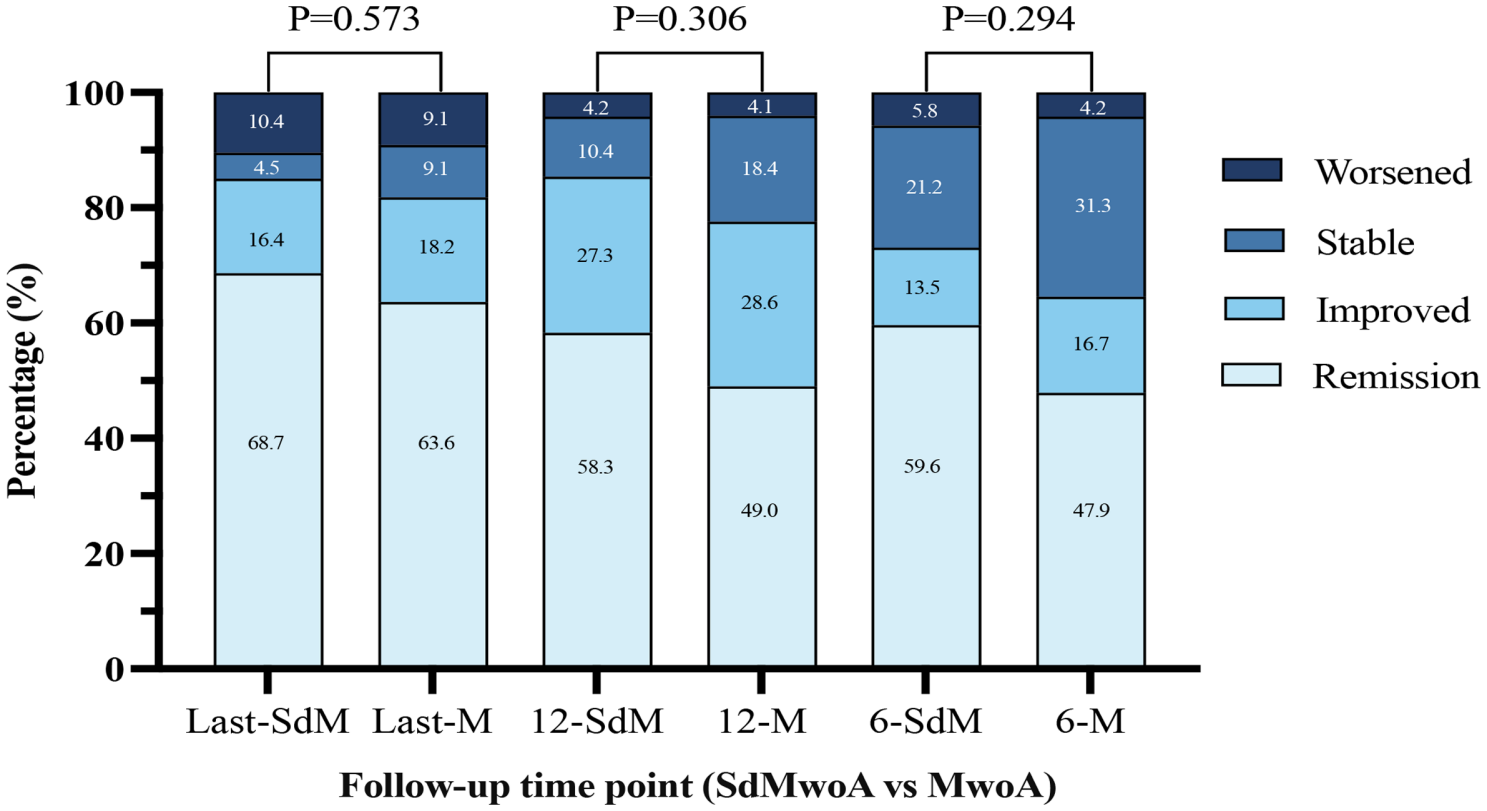
Distribution of headache outcomes at 6 months, 12 months, and the last follow-up by attack-duration group.

The follow-up completeness and overall headache outcomes at each time point are shown in Table 2 and Figure 2. At 6 months, follow-up completion rates were similar between the SdMwoA and MwoA groups (63.4% vs. 62.3%, *p* = 0.888), and no significant differences were observed at 12 months (55.8% vs. 61.3%, *p* = 0.478) or at the last follow-up (69.1% vs. 69.5%, *p* = 0.952). The median follow-up duration was 25 months [interquartile range (IQR) 18–39] in the SdMwoA group and 26 months (IQR 16–42) in the MwoA group (*p* = 0.601), indicating broadly comparable long-term follow-up quality across groups.

**Table 2:** Follow-up completion and overall improvement.

Outcome	SdMwoA (*n* = 82)	MwoA (*n* = 77)	*p*-value
6-month follow-up			
Completed follow-up, *n* (%)	52 (63.4%)	48 (62.3%)	0.888
Any improvement^a^, *n* (%)	38 (73.1%)	31 (64.6%)	0.359
Any improvement, LTFU^b^ = improvement, *n* (%)	68 (82.9%)	60 (77.9%)	0.426
Any improvement, LTFU = no improvement, *n* (%)	38 (46.3%)	31 (40.3%)	0.439

	SdMwoA (*n* = 86)	MwoA (*n* = 80)	*p*-value
12-month follow-up			
Completed follow-up, *n* (%)	48 (55.8%)	49 (61.3%)	0.478
Any improvement, *n* (%)	41 (85.4%)	38 (77.6%)	0.319
Any improvement, LTFU = improvement, *n* (%)	79 (91.9%)	69 (86.3%)	0.245
Any improvement, LTFU = no improvement, *n* (%)	41 (47.7%)	38 (47.5%)	0.982

	SdMwoA (*n* = 97)	MwoA (*n* = 95)	*p*-value
Last follow-up			
Completed follow-up, *n* (%)	67 (69.1%)	66 (69.5%)	0.952
Median follow-up duration, months median (IQR)	25 (18, 39)	26 (16, 42)	0.601
Any improvement, *n* (%)	57 (85.1%)	54 (81.8%)	0.613
Any improvement, LTFU = improvement, *n* (%)	87 (89.7%)	83 (87.4%)	0.613
Any improvement, LTFU = no improvement, *n* (%)	57 (58.8%)	54 (56.8%)	0.778

a Any improvement: remission or improved.

b LTFU: lost to follow-up.

With regard to headache outcomes, when remission and improved were combined, both groups showed high and comparable rates of improvement at all time points. At 6 months, the proportions of patients with any improvement were 73.1% in the SdMwoA group and 64.6% in the MwoA group; at 12 months, the proportions of patients with any improvement were 85.4 and 77.6%, respectively; and at the last follow-up, the proportions of patients with any improvement were 85.1 and 81.8%, respectively. None of these differences reached statistical significance. As illustrated in Figure 2, more than 60% of the children in each group had achieved a completely headache-free state by the last follow-up, approximately 20% showed some improvement relative to baseline, and only a small minority had stable or worsened headache. The distribution of the four ordinal outcome categories (remission, improved, stable, and worsened) did not differ significantly between groups at any time point.

### 3.4 Factors associated with headache remission and improvement

**Figure 3:**
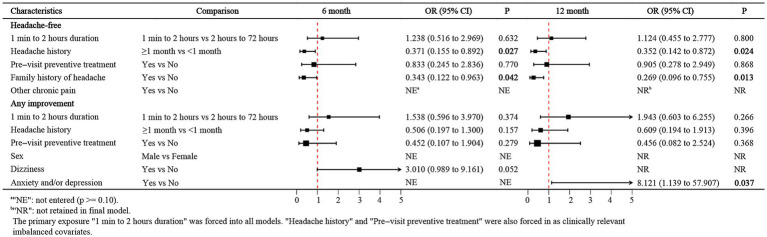
Forest plot of prognostic factors for headache-free status and any improvement at 6 and 12 months.

The multivariable logistic regression results are presented in Figure 3. When complete headache remission was used as the outcome, attack duration was not significantly associated with remission at either 6 or 12 months (6 months: OR = 1.238; 12 months: OR = 1.124; both *p* > 0.05). In contrast, children with a headache history ≥1 month before presentation were substantially less likely to achieve a headache-free state at both time points (6 months: OR = 0.371, 95% CI 0.155–0.892; 12 months: OR = 0.352, 95% CI 0.142–0.872), and those with a family history of headache also had lower odds of remission (6 months: OR = 0.343, 95% CI 0.122–0.963; 12 months: OR = 0.269, 95% CI 0.096–0.755). Pre-visit preventive treatment before the index visit was not significantly associated with remission at either time point.

When the outcome was defined as any improvement, attack duration, headache duration, and previous treatment similarly did not have consistent independent effects (Figure 3). At 6 months, children who reported vertigo symptoms had a somewhat greater likelihood of any improvement (OR = 3.010, 95% CI 0.99–9.16; *p* = 0.052), with borderline statistical significance. At 12 months, self-reported anxiety and/or depression was associated with a greater probability of any improvement (OR = 8.121, 95% CI 1.139–57.907; *p* = 0.037).

### 3.5 Time to achieve a headache-free state

**Figure 4:**
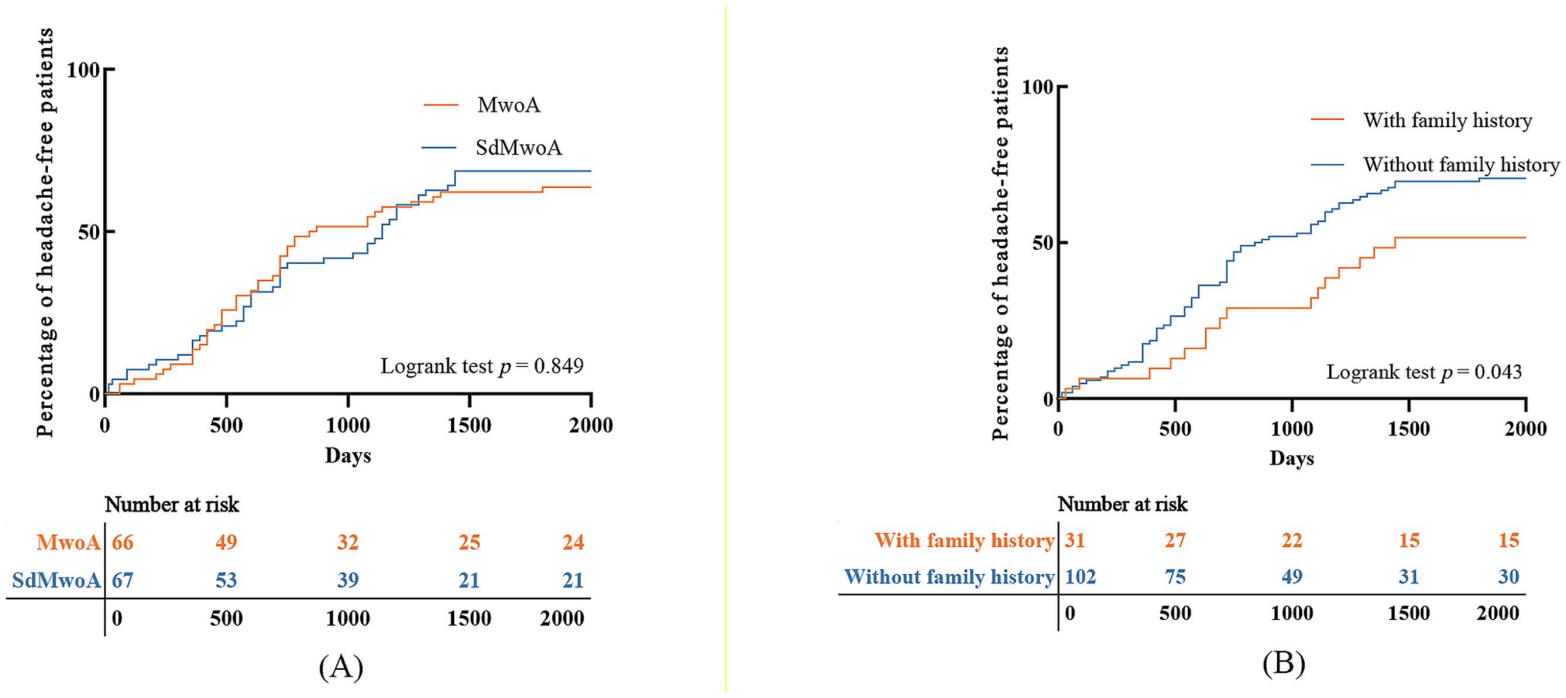
Kaplan–Meier analysis of time to headache-free status. **(A)** Comparison between the SdMwoA and MwoA groups; **(B)** comparison between patients with and without a family history of headache.

In the early follow-up phase, the cumulative proportion of headache-free children in both groups increased rapidly but then gradually plateaued, with more than half of the patients achieving a headache-free state by the end of follow-up (Figure 4A). The two survival curves almost overlapped throughout the observation period, and the log-rank test revealed no significant difference between the groups (*p* = 0.849).

When stratified by family history of headache (Figure 4B), children without a family history had a distinctly higher cumulative probability of becoming headache-free than those with a family history did. The two curves separated progressively from the early follow-up period onward, and the difference persisted until the end of follow-up (log-rank *p* = 0.043).

In the sensitivity analyses, re-estimating the models under extreme assumptions for patients lost to follow-up and subdividing the short-duration group into ≥1 min and <30 min and ≥30 min and <2 h strata did not substantially change the estimates, and the Kaplan–Meier curves for all three duration strata largely overlapped (Supplementary Tables 1–3 and Supplementary Figures 1, 2).

## 4 Discussion

This study addressed the clinical controversy of whether patients with pediatric migraine without aura must meet the 2-h minimum attack duration specified by the ICHD-3. Using real-world data from consecutive patients at a single pediatric headache clinic, we compared SdMwoA patients with MwoA patients across multiple follow-up time points. We found no significant differences between the two groups in the proportions of patients who achieved headache remission or any improvement at 6 months, 12 months, or the last follow-up (Table 2 and Figure 2), and the Kaplan–Meier curves for time to a headache-free state were almost superimposed (Figure 4A). These findings suggest that an attack duration of <2 h alone is insufficient to exclude children from the category of pediatric MwoA. Rather, such attacks may be better viewed as short-duration variants within the migraine spectrum instead of as distinct primary headaches with fundamentally different prognoses. Notably, this interpretation was further supported by prespecified sensitivity analyses, including extreme-assumption analyses for loss to follow-up and duration-stratified analyses, which yielded consistent results.

Previous work has shown that migraine attacks in children are generally shorter than those in adults and that the strict application of the adult-derived 2-h lower limit markedly reduces diagnostic sensitivity, whereas including attacks lasting 1–2 h substantially improves case detection (10–12). In this context, longitudinal studies have also suggested that pediatric headache phenotypes may evolve over time and that diagnostic categories can overlap or shift, which supports a cautious interpretation of short-duration presentations within the broader migraine spectrum (13–15). Our study extends this literature by providing outcome-based evidence. Among children with a typical migraine phenotype and attack duration of 1 min to 2 h, baseline characteristics, remission rates, proportions with any improvement, and the time course to a headache-free state were highly comparable to those of children with MwoA whose attacks lasted 2–72 h.

A related concern is whether short-duration attacks might be confused with TTH (16), as pediatric TTH can also present with short- to medium-duration, recurrent headache episodes and may share some clinical features with migraine (17). Nonetheless, our findings favor classifying attacks shorter than 2 h as migraine when several key conditions are met. First, most children in our cohort reported associated symptoms such as nausea and vomiting, which have high discriminative value for migraine and are not typical of TTH (3). Second, we observed that a positive family history of headache was associated with a lower likelihood of remission; this novel observation is in accordance with previous evidence that primary headaches in children, particularly migraine, are markedly more familial and that a higher familial headache burden is related to earlier onset and more severe or persistent disease (18). Therefore, our conclusions should not be interpreted as suggesting that all headaches lasting <2 h should be diagnosed as migraine. Instead, we emphasize that when clinical phenotype, associated symptoms, and family history are strongly suggestive of migraine, children should not be excluded from the diagnosis of MwoA solely because their typical attack duration does not reach 2 h; clinicians should integrate all migraine-oriented features and make a comprehensive judgment.

Our data further indicate that the key determinants of prognosis in this cohort were not the duration of individual attacks but the interval from onset to first specialist consultation and the presence of a family history of headache. Multivariate logistic regression revealed that children with a headache history of ≥1 month before presentation were significantly less likely to achieve a headache-free state at both 6 and 12 months, suggesting that a longer prediagnosis course is associated with a lower probability of short- to mid-term remission. These findings differ from those of some observational studies that focused on very short-term outcomes of more than 1–3 months (19), possibly because our longer follow-up window captured the cumulative impact of the disease course more fully. In addition, a positive family history of headache was consistently associated with a higher risk of persistent or recurrent headaches at multiple time points, and Kaplan–Meier analysis revealed a markedly lower cumulative probability of becoming headache-free in children with a family history than in those without, with separation of survival curves from the early follow-up period onward. This finding is in line with long-term adolescent follow-up studies (20) and supports the notion that genetic susceptibility may influence not only the occurrence of migraine but also the pace of symptom resolution.

With respect to the secondary outcome of any improvement, we observed a positive association between self-reported emotional or anxiety problems and clinical benefit, which contrasts with previous work showing that depressive and anxiety symptoms predict an unfavorable short-term prognosis in pediatric patients with migraine (19). In our view, this pattern is more plausibly explained by greater help-seeking behavior and better adherence to treatment and follow-up among affected children and their families rather than by a genuinely protective effect of emotional symptoms on headache outcomes.

Because of differences in the study population, diagnostic procedures, follow-up methods, and observation periods, our outcomes cannot be directly equated with those reported in all previous studies. Overall, however, our results are consistent with several pediatric headache follow-up studies, which have shown that most children with migraine improve over short- to medium-term follow-up, with approximately 60–80% achieving remission or a marked reduction in headache burden (19, 21, 22). In contrast, the long-term prognosis of childhood headache appears much less favorable: cohort studies with 10–40 years of follow-up suggest that only approximately one quarter to one third of affected individuals become completely headache-free, whereas the majority continue to experience headaches into adulthood (13–15, 23, 24).

This study has several unavoidable limitations inherent to its single-center retrospective cohort design. First, despite sensitivity analyses under two extreme assumptions, the moderate loss-to-follow-up rate may still introduce bias. Because attrition may be non-random and outcomes among patients lost to follow-up may differ systematically from those retained, residual bias cannot be excluded. Accordingly, prospective studies with larger sample sizes and more complete follow-up are needed to validate these findings, and small between-group differences could have been missed due to limited statistical power related to the sample size and attrition, which increases the likelihood of a type II error. Second, some children were very young, had brief attacks, or first presented after their symptoms had already resolved, which may have led to an underestimation of headache burden and information bias. Third, because migraine and tension-type headache share overlapping clinical features (25) and can transform into one another over time (14), misclassification cannot be fully ruled out despite our efforts to exclude children with a typical TTH phenotype, and residual confounding remains possible. In addition, pre-visit preventive regimens were heterogeneous and could not be fully standardized or quantified, which may have contributed to residual confounding.

In summary, real-world follow-up data from this cohort indicate that, among children with migraine without aura, differences in single-attack duration between 1 min to 2 h and 2 h to 72 h were not significantly associated with headache remission or any improvement at 6 or 12 months or at last follow-up, and attack duration did not emerge as an independent prognostic factor. In contrast, a longer interval from onset to first consultation and a positive family history of headache were associated with lower remission rates and delayed achievement of a headache-free state and thus appeared to be more important prognostic factors in this cohort.

From a clinical perspective, these findings support a child-centered and flexible interpretation of the ICHD-3 duration criterion for pediatric migraine without aura. When the migraine phenotype is clearly established, clinicians should avoid excluding a diagnosis of MwoA solely on the basis of an attack duration <2 h. Future epidemiological and diagnostic-criteria studies should include children with typical migraine features and an attack duration ≥1 min but <2 h as a distinct duration stratum to accumulate further outcome-oriented evidence specific to pediatric populations.

## Data Availability

The datasets presented in this article are not readily available because the dataset contains clinical information derived from routine care and includes potentially identifiable patient data. Therefore, it cannot be made publicly available due to ethical and legal restrictions and the conditions of the institutional ethics approval. De-identified data may be made available from the corresponding author upon reasonable request and with approval from the institutional review board/ethics committee. Requests to access the datasets should be directed to dr_jiangli@hotmail.com.

## References

[1] Na JH. Application and effectiveness of dietary therapies for pediatric migraine. Headache Pain Res. (2024) 25:34–41. doi: 10.62087/hpr.2024.0007

[2] Onofri A Pensato U Rosignoli C Wells-Gatnik W Stanyer E Ornello R . Primary headache epidemiology in children and adolescents: a systematic review and meta-analysis. J Headache Pain. (2023) 24:8. doi: 10.1186/s10194-023-01541-0, 36782182 PMC9926688

[3] Headache Classification Committee of the International Headache Society (IHS). The international classification of headache disorders, 3rd edition. Cephalalgia. (2018) 38:1–211. doi: 10.1177/0333102417738202 29368949

[4] Özge A Faedda N Abu-Arafeh I Gelfand AA Goadsby PJ Cuvellier JC . Experts’ opinion about the primary headache diagnostic criteria of the ICHD-3rd edition beta in children and adolescents. J Headache Pain. (2017) 18:109. doi: 10.1186/s10194-017-0818-y, 29285570 PMC5745373

[5] Lima MM Bazan R Martin LC Martins AS Luvizutto GJ Betting LE . Critical analysis of diagnostic criteria (ICHD-3 beta) about migraine in childhood and adolescence. Arq Neuropsiquiatr. (2015) 73:1005–8. doi: 10.1590/0004-282x20150162 26465286

[6] Patterson Gentile C Hershey AD Szperka CL. A critical appraisal of the international classification of headache disorders migraine diagnostic criteria based on a retrospective multicenter cross-sectional headache registry study in youth. Headache. (2024) 64:1217–29. doi: 10.1111/head.14858, 39463026 PMC11560481

[7] Francis MV. Brief migraine episodes in children and adolescents-a modification to international headache society pediatric migraine (without aura) diagnostic criteria. Springerplus. (2013) 2:77. doi: 10.1186/2193-1801-2-77, 23526480 PMC3602609

[8] Khan A Liu S Tao F. Current trends in pediatric migraine: clinical insights and therapeutic strategies. Brain Sci. (2025) 15:280. doi: 10.3390/brainsci15030280, 40149800 PMC11940401

[9] Hershey AD Powers SW Vockell AL LeCates S Kabbouche MA Maynard MK. PedMIDAS: development of a questionnaire to assess disability of migraines in children. Neurology. (2001) 57:2034–9. doi: 10.1212/wnl.57.11.2034, 11739822

[10] Abu-Arafeh I Gelfand AA. The childhood migraine syndrome. Nat Rev Neurol. (2021) 17:449–58. doi: 10.1038/s41582-021-00497-6, 34040231

[11] Abu-Arafeh I Callaghan M. Short migraine attacks of less than 2 h duration in children and adolescents. Cephalalgia. (2004) 24:333–8. doi: 10.1111/j.1468-2982.2004.00670.x, 15096221

[12] Papetti L Salfa I Battan B Moavero R Termine C Bartoli B . Features of primary chronic headache in children and adolescents and validity of ICHD 3 criteria. Front Neurol. (2019) 10:92. doi: 10.3389/fneur.2019.00092, 30890994 PMC6413701

[13] Brna P Dooley J Gordon K Dewan T. The prognosis of childhood headache: a 20-year follow-up. Arch Pediatr Adolesc Med. (2005) 159:1157–60. doi: 10.1001/archpedi.159.12.1157 16330740

[14] Dooley JM Augustine HF Brna PM Digby AM. The prognosis of pediatric headaches--a 30-year follow-up study. Pediatr Neurol. (2014) 51:85–7. doi: 10.1016/j.pediatrneurol.2014.02.022, 24814057

[15] Lopes CFR Dos Santos TP Martins IP. Prognosis of headache in children: a 25-year follow-up. Childs Nerv Syst. (2022) 38:619–26. doi: 10.1007/s00381-021-05420-4, 35059785

[16] Genizi J Khourieh Matar A Schertz M Zelnik N Srugo I. Pediatric mixed headache—the relationship between migraine, tension-type headache and learning disabilities—in a clinic-based sample. J Headache Pain. (2016) 17:42. doi: 10.1186/s10194-016-0625-x, 27102119 PMC4840135

[17] Baglioni V Orecchio S Esposito D Faedda N Natalucci G Guidetti V. Tension-type headache in children and adolescents. Life. (2023) 13:825. doi: 10.3390/life13030825, 36983980 PMC10056425

[18] Winther CCH Berring-Uldum AA Debes NM. Inheritance of primary headache in children and adolescents—a scoping review. Neuropediatrics. (2025) 56:152–9. doi: 10.1055/a-2505-8261, 39701164

[19] Orr SL Turner A Kabbouche MA Horn PS O’Brien HL Kacperski J . Predictors of short-term prognosis while in pediatric headache care: an observational study. Headache. (2019) 59:543–55. doi: 10.1111/head.13477, 30671933 PMC6459721

[20] Monastero R Camarda C Pipia C Camarda R. Prognosis of migraine headaches in adolescents: a 10-year follow-up study. Neurology. (2006) 67:1353–6. doi: 10.1212/01.wnl.0000240131.69632.4f, 17060559

[21] Guidetti V Galli F. Evolution of headache in childhood and adolescence: an 8-year follow-up. Cephalalgia. (1998) 18:449–54. doi: 10.1046/j.1468-2982.1998.1807449.x, 9793696

[22] Kienbacher C Wöber C Zesch HE Hafferl-Gattermayer A Posch M Karwautz A . Clinical features, classification and prognosis of migraine and tension-type headache in children and adolescents: a long-term follow-up study. Cephalalgia. (2006) 26:820–30. doi: 10.1111/j.1468-2982.2006.01108.x, 16776697

[23] Bille B. A 40-year follow-up of school children with migraine. Cephalalgia. (1997) 17:488–91. doi: 10.1046/j.1468-2982.1997.1704488.x, 9209767

[24] Termine C Ferri M Livetti G Beghi E Salini S Mongelli A . Migraine with aura with onset in childhood and adolescence: long-term natural history and prognostic factors. Cephalalgia. (2010) 30:674–81. doi: 10.1177/0333102409351803, 20511205

[25] Albers L Straube A Landgraf MN Filippopulos F Heinen F von Kries R. Migraine and tension type headache in adolescents at grammar school in Germany—burden of disease and health care utilization. J Headache Pain. (2015) 16:534. doi: 10.1186/s10194-015-0534-4, 26055241 PMC4467810

