# Supplementary material for: Short-duration migraine without aura in children: a retrospective cohort study of attack duration and prognosis
Source: Front Neurol. 2026 Mar 10;17:1782182. doi: 10.3389/fneur.2026.1782182 (PMC13008621; doi:10.3389/fneur.2026.1782182)
Supplement: Supplementary file 1 [file Table_1.docx]

**Supplementary Table 1** Baseline characteristics among three groups.

| **Variables** | **vSdMwoA ^a^ (n=41)** | **hSdMwoA ^b^ (n=56)** | **MwoA (n=95)** | ***P*-value** |
| --- | --- | --- | --- | --- |
| **Sex, n (%)** |  |  |  | 0.628 |
| Female | 19 (46.3%) | 21 (37.5%) | 42 (44.2%) |  |
| Male | 22 (53.7%) | 35 (62.5%) | 53 (55.8%) |  |
| **Age, years, median (IQR)** | 9.0 (8.0, 11.0) | 10.0 (8.0, 11.0) | 10.0 (8.0, 12.0) | 0.205 |
| **Headache history ≥1 month, n (%)** | 21 (51.2%) | 19 (33.9%) | 58 (61.1%) | **0.006** |
| **Pre-visit preventive treatment, n (%)** | 6 (14.6%) | 6 (10.7%) | 28 (29.5%) | **0.013** |
| **Family history of headache, n (%)** | 5 (12.2%) | 15 (26.8%) | 22 (23.2%) | 0.209 |
| **Dizziness, n (%)** | 8 (19.5%) | 18 (32.1%) | 36 (37.9%) | 0.109 |
| **Other chronic pain, n (%)** | 15 (36.6%) | 17 (30.4%) | 29 (30.5%) | 0.757 |
| **Self-rated anxiety and/or depression, n (%)** | 4 (9.8%) | 6 (10.7%) | 7 (7.4%) | 0.752 |
| **Pain laterality, n (%)** |  |  |  | 0.813 |
| Bilateral | 17 (41.5%) | 26 (46.4%) | 45 (47.4%) |  |
| Unilateral | 24 (58.5%) | 30 (53.6%) | 50 (52.6%) |  |
| **Pain location, n (%)** |  |  |  | 0.074 |
| Frontal | 15 (36.6%) | 15 (26.8%) | 16 (16.8%) |  |
| Mixed | 13 (31.7%) | 24 (42.9%) | 45 (47.4%) |  |
| Occipital | 2 (4.9%) | 1 (1.8%) | 5 (5.3%) |  |
| Temporal | 10 (24.4%) | 8 (14.3%) | 23 (24.2%) |  |
| Vertex | 1 (2.4%) | 8 (14.3%) | 6 (6.3%) |  |
| **Pain quality, n (%)** |  |  |  | 0.880 |
| Other | 32 (78.0%) | 46 (82.1%) | 76 (80.0%) |  |
| Throbbing | 9 (22.0%) | 10 (17.9%) | 19 (20.0%) |  |
| **Pain frequency, n (%)** |  |  |  | 0.494 |
| <1/month | 2 (4.9%) | 3 (5.4%) | 4 (4.2%) |  |
| 1-3/month | 3 (7.3%) | 2 (3.6%) | 6 (6.3%) |  |
| 1/week | 2 (4.9%) | 4 (7.1%) | 8 (8.4%) |  |
| 2-6/week | 9 (22.0%) | 7 (12.5%) | 19 (20.0%) |  |
| Daily | 25 (61.0%) | 40 (71.4%) | 58 (61.1%) |  |
| **Pain intensity, n (%)** |  |  |  | **0.025** |
| Mild | 10 (24.4%) | 4 (7.1%) | 8 (8.4%) |  |
| Moderate or Severe | 31 (75.6%) | 52 (92.9%) | 87 (91.6%) |  |
| **PedMIDAS ^c^, n (%)** |  |  |  | 0.208 |
| Little to none | 10 (24.4%) | 6 (10.7%) | 7 (7.4%) |  |
| Mild | 14 (34.1%) | 22 (39.3%) | 40 (42.1%) |  |
| Moderate | 7 (17.1%) | 11 (19.6%) | 17 (17.9%) |  |
| Severe | 10 (24.4%) | 17 (30.4%) | 31 (32.6%) |  |

^a^ “vSdMwoA”: patients who met all MwoA criteria except attack duration, with a typical attack duration ≥ 1 minute and < 30 minutes.

^b^ “hSdMwoA”: patients who met all MwoA criteria except attack duration, with a typical attack duration ≥ 30 minutes and < 2 hours.

^c^ “PedMIDAS”: Pediatric Migraine Disability Assessment Scale graded as 0–10 (little to none), 11–30 (mild), 31–50 (moderate), and > 50 (severe).

**Supplementary Table 2** Follow-up completion and overall improvement among three groups.

| **Outcome** | **vSdMwoA** ^a^ | **hSdMwoA** ^b^ | **MwoA** | ***P*-value** |
| --- | --- | --- | --- | --- |
| **6-month follow-up** | n=36 | n=46 | n=77 |  |
| Completed follow-up, n (%) | 25 (69.4%) | 27 (58.7%) | 48 (62.3%) | 0.601 |
| Headache status, n (%) |  |  |  | 0.558 |
| Remission | 16 (64.0%) | 15 (55.6%) | 23 (47.9%) |  |
| Improved | 2 (8.0%) | 5 (18.5%) | 8 (16.7%) |  |
| Stable | 5 (20.0%) | 6 (22.2%) | 15 (31.3%) |  |
| Worsened | 2 (8.0%) | 1 (3.7%) | 2 (4.2%) |  |
| Any improvement ^c^, n (%) | 18 (72.0%) | 20 (74.1%) | 31 (64.6%) | 0.648 |
| Any improvement, LTFU ^d^= improvement, n (%) | 29 (80.6%) | 39 (84.8%) | 60 (77.9%) | 0.649 |
| Any improvement, LTFU = no improvement, n (%) | 18 (50.0%) | 20 (43.5%) | 31 (40.3%) | 0.623 |
| **12-month follow-up** | n=38 | n=48 | n=80 |  |
| Completed follow-up, n (%) | 23 (60.5%) | 25 (52.1%) | 49 (61.3%) | 0.478 |
| Headache status, n (%) |  |  |  | 0.589 |
| Remission | 14 (60.9%) | 14 (56.0%) | 24 (49.0%) |  |
| Improved | 5 (21.7%) | 8 (32.0%) | 14 (28.6%) |  |
| Stable | 3 (13.0%) | 2 (8.0%) | 9 (18.4%) |  |
| Worsened | 1 (4.3%) | 1 (4.0%) | 2 (4.1%) |  |
| Any improvement, n (%) | 19 (82.6%) | 22 (88.0%) | 38 (77.6%) | 0.543 |
| Any improvement, LTFU = improvement, n (%) | 34 (89.5%) | 45 (93.8%) | 69 (86.3%) | 0.417 |
| Any improvement, LTFU = no improvement, n (%) | 19 (50.0%) | 22 (45.8%) | 38 (47.5%) | 0.929 |
| **Last follow-up** | n=41 | n=56 | n=95 |  |
| Completed follow-up, n (%) | 29 (70.7%) | 38 (67.9%) | 66 (69.5%) | 0.953 |
| Median follow-up duration, months Median (IQR) | 24 (18,36) | 27 (17,43) | 26 (16,42) | 0.632 |
| Headache status, n (%) |  |  |  | 0.512 |
| Remission | 18 (62.1%) | 28 (73.7%) | 42 (63.6%) |  |
| Improved | 5 (17.2%) | 6 (15.8%) | 12 (18.2%) |  |
| Stable | 3 (10.3%) | 0 (0.0%) | 6 (9.1%) |  |
| Worsened | 3 (10.3%) | 4 (10.5%) | 6 (9.1%) |  |
| Any improvement, n (%) | 23 (79.3%) | 34 (89.5%) | 54 (81.8%) | 0.476 |
| Any improvement, LTFU = improvement, n (%) | 35 (85.4%) | 52 (92.9%) | 83 (87.4%) | 0.457 |
| Any improvement, LTFU = no improvement, n (%) | 23 (56.1%) | 34 (60.7%) | 54 (56.8%) | 0.870 |

^a^ “vSdMwoA”: patients who met all MwoA criteria except attack duration, with a typical attack duration ≥ 1 minute and < 30 minutes.

^b^ “hSdMwoA”: patients who met all MwoA criteria except attack duration, with a typical attack duration ≥ 30 minutes and < 2 hours.

^c^ “Any improvement”: remission or improved. Percentages for ‘Any improvement’ are calculated among participants with completed follow-up at that time point.

^d^ “LTFU”: lost to follow-up.

**Supplementary Table 3** Sensitivity analyses for predictors of headache-free status and any improvement under extreme follow-up assumptions

| **Variable** | **6-month OR (95%CI), *P*-value** | | **12-month OR (95%CI), *P*-value** | |
| --- | --- | --- | --- | --- |
| **Headache-free** | **Sens A^a^** | **Sens B ^b^** | **Sens A** | **Sens B** |
| 1 min to 2 hours duration | 1.363(0.649,2.865), 0.414 | 0.999(0.482,2.070), 0.998 | 1.435(0.658,3.127), 0.364 | 0.749(0.357,1.569), 0.443 |
| Headache history ≥1 month | 0.794(0.369,1.709), 0.556 | 0.297(0.145,0.607), **<0.001** | 0.806(0.361,1.800), 0.599 | 0.268(0.128,0.561), **<0.001** |
| Pre-visit preventive treatment | 0.575(0.208,1.591), 0.287 | 1.269(0.489,3.296), 0.625 | 0.979(0.352,2.724), 0.968 | 1.066(0.424,2.679), 0.892 |
| Family history of headache | 0.391(0.170,0.899), **0.027** | NR ^d^ | 0.299(0.127,0.705), **0.006** | 0.436(0.174,1.096), 0.078 |
| Other chronic pain | NE ^c^ | NE | 0.255(0.119,0.547), **<0.001** | NR |
| **Any improvement** | **Sens C ^e^** | **Sens D ^f^** | **Sens C** | **Sens D** |
| 1 min to 2 hours duration | 1.737(0.732,4.125), 0.210 | 0.916(0.457,1.837), 0.806 | 1.892(0.656,5.456), 0.238 | 0.784(0.395,1.558), 0.488 |
| Headache history ≥1 month | 0.877(0.362,2.120), 0.770 | 0.307(0.153,0.615), **<0.001** | 0.869(0.295,2.565), 0.800 | 0.349(0.175,0.694), 0.003 |
| Pre-visit preventive treatment | 0.360(0.096,1.353), 0.130 | 1.155(0.473,2.820), 0.751 | 0.424(0.088,2.052), 0.286 | 0.998(0.422,2.361), 0.996 |
| Male | NE | NE | 2.557(0.925,7.073), 0.070 | NR |
| Dizziness | 3.198(1.117,9.153), **0.030** | NR | NR | NR |
| Self-rated anxiety and/or depression | NE | NE | NR | 6.722(1.423,31.755), 0.016 |

^a^ “Sens A”: all lost to follow-up imputed as headache-free.

^b^ “Sens B”: all lost to follow-up imputed as not headache-free.

^c^ “NE”: not entered (p≥0.10 in univariable).

^d^ “NR”: not retained in final model.

^e^ “Sens C”: all lost to follow-up imputed as any improvement.

^f^ “Sens D”: all lost to follow-up imputed as no improvement.
