# Supplementary figures and images for: Short-duration migraine without aura in children: a retrospective cohort study of attack duration and prognosis
Source: Front Neurol. 2026 Mar 10;17:1782182. doi: 10.3389/fneur.2026.1782182 (PMC13008621; doi:10.3389/fneur.2026.1782182)

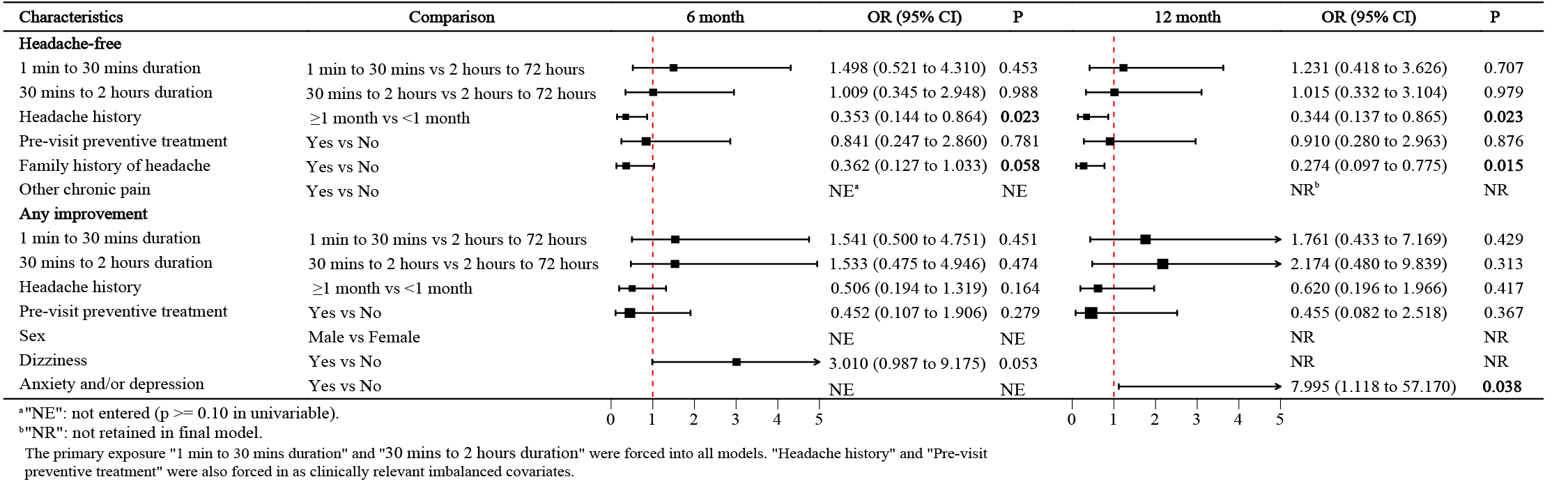

Supplement: Supplementary file 2 [file Image_1.tif]

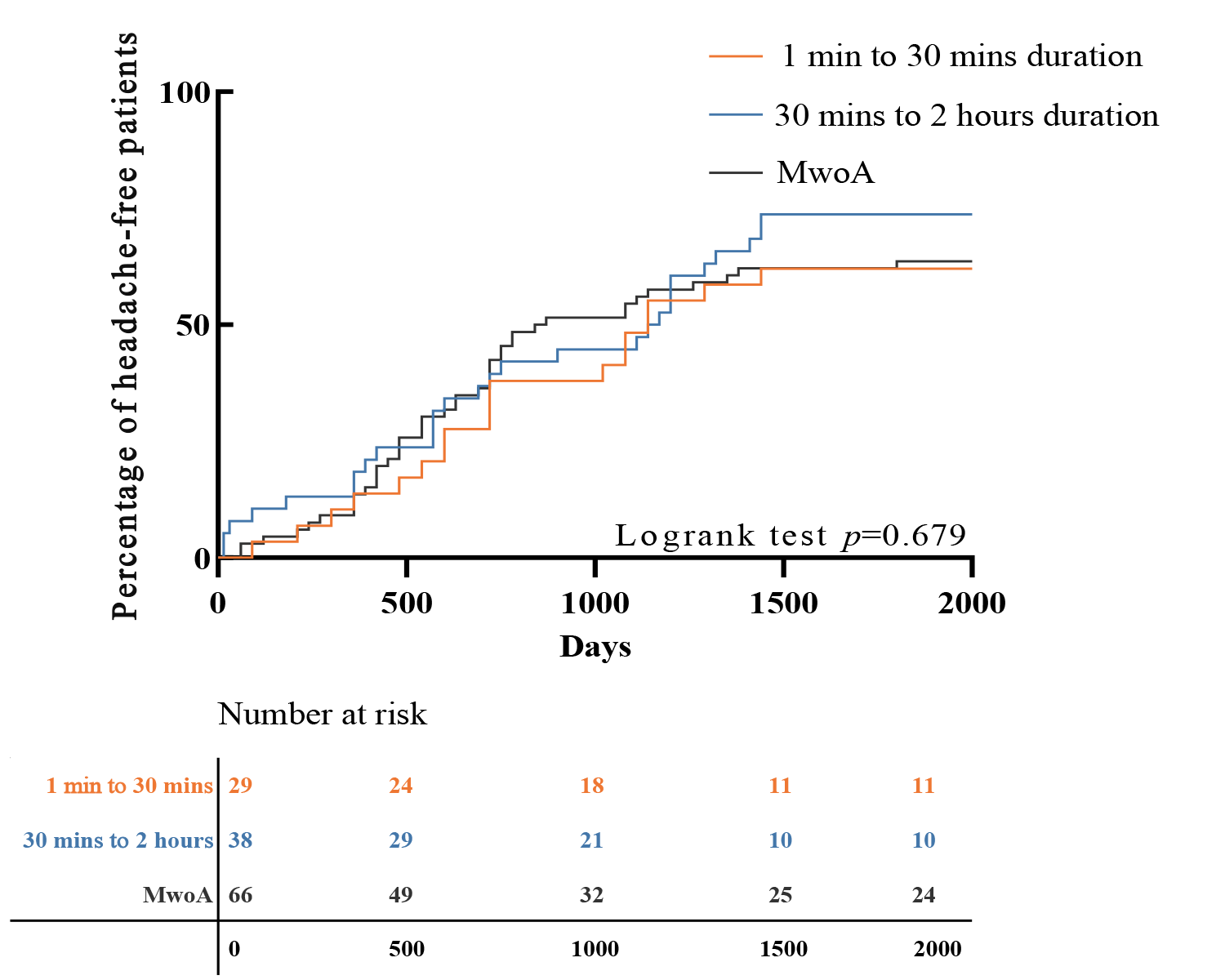

Supplement: Supplementary file 3 [file Image_2.tif]
